# Editorial: Skeletal muscle—From developmental concepts to therapy

**DOI:** 10.3389/fcell.2022.1111561

**Published:** 2023-01-12

**Authors:** Susanne Dietrich, Frank Richard Schubert, Chrissa Kioussi

**Affiliations:** ^1^ Institute for Biomedical and Biomolecular Science (IBBS), School of Pharmacy and Biomedical Sciences, University of Portsmouth, Portsmouth, United Kingdom; ^2^ Institute of Biomedical and Biomolecular Sciences, University of Portsmouth, Portsmouth, United Kingdom; ^3^ Department of Pharmaceutical Sciences, College of Pharmacy Oregon State University, Corvallis, OR, United States

**Keywords:** muscle, disease, therapy, injury, regeneration

## Introduction

Skeletal or voluntary muscle is crucial for locomotion, maintenance of posture and balance, respiration, control over the sense organs and food uptake and thus, is essential for life. Skeletal muscle also plays key roles for thermoregulation and metabolism. Skeletal muscle is the most adaptable organ in the body, owing to its cellular plasticity and high regenerative capacity. However, muscle disorders occur due to genetic mutations or may be an indirect consequence of neurological and metabolic malfunctions, injuries, cachexia, and aging. Skeletal muscle diseases are associated with muscle weakness, impaired motility, and in some diseases, fatal muscle degeneration. Therefore, novel strategies to ameliorate or, better, cure these diseases are actively sought. This Research Topic of Frontiers in Developmental and Cell Biology, that includes two review articles and three research articles, is dedicated to genetic mutations, injury and aging of the skeletal muscle, and links research aimed at understanding regulatory mechanisms controlling muscle development and function with research aimed at developing therapy.

## Genetic mutations

### Duchenne

**FIGURE 1:**
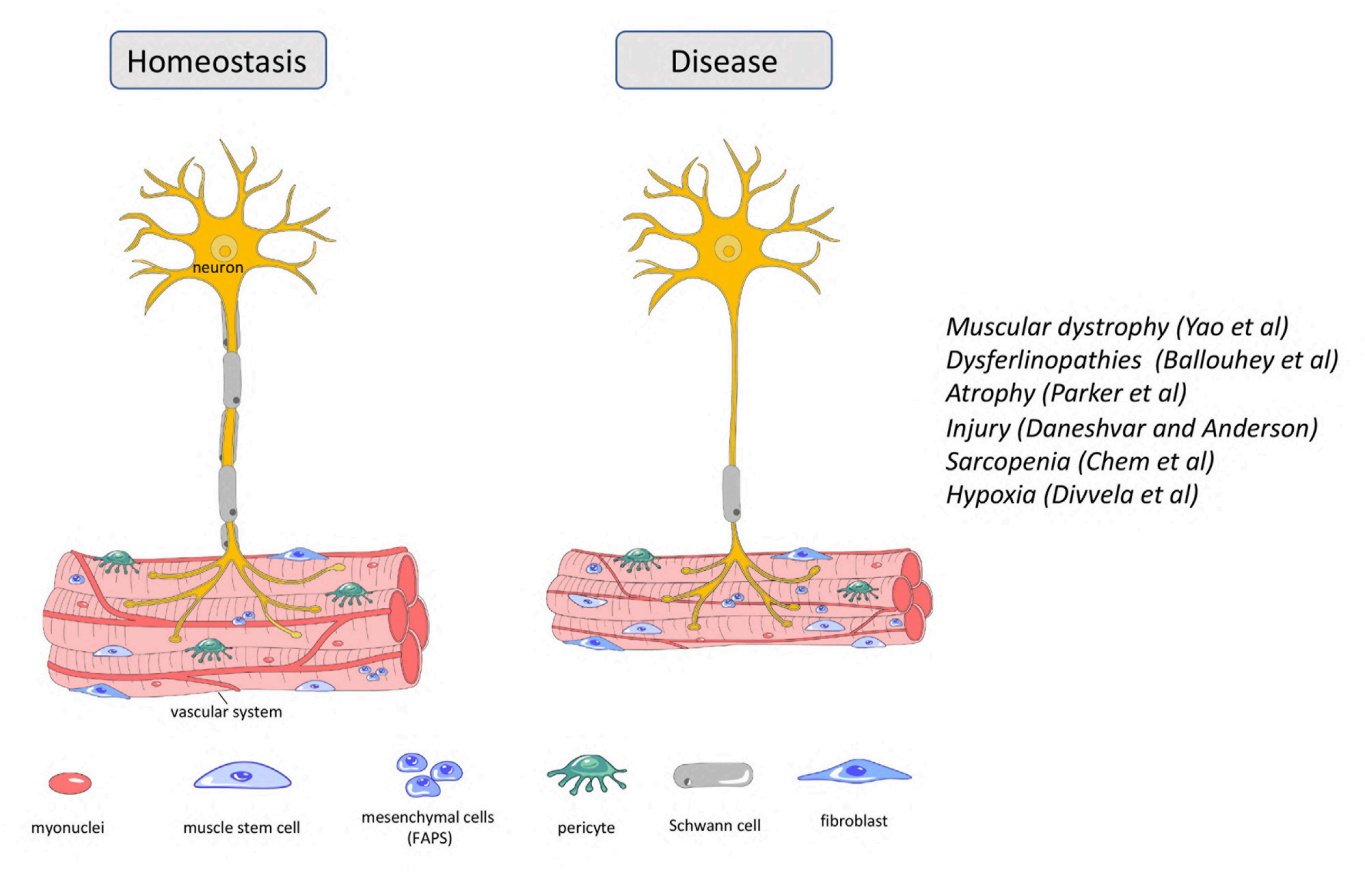
Cellular content of neuron and skeletal muscle during homeostasis and disease state.

Muscular dystrophies are inherited diseases that directly affect skeletal muscle. Symptoms range from mild to very severe with respiratory or cardiac failure. Duchenne muscular dystrophy (DMD) is a lethal, X-linked neuromuscular disorder; it is the most common muscular dystrophy affecting 1:3,500–5,000 boys. The disease results from mutations in the *DYSTROPHIN* (*DMD)* gene. Here in this Research Topic, Yao et al. discussed the current therapeutic strategies for *DMD* that have entered the clinical phase and/or have great potential for clinical translation. Therapeutic strategies targeting *DMD* such as exon skipping, stop-codon read-through, gene addition and gene editing have the potential to provide long-lasting and one-time treatment for DMD. Cell-/stem cell-based therapies are also being tested but have not entered the clinic since the generation of the most suitable cell type, cell delivery and safety issues have not been resolved (Sun et al., 2020). Therapeutic strategies acting on downstream pathological mechanisms of DMD include antifibrotic and anti-inflammatory interventions in addition to managing calcium dysregulation, oxidative stress, ischemia, atrophy, and bone homeostasis (Figure 1). The authors performed a meta-analysis of gene profiling in DMD patients and identified new gene–disease associations and novel therapeutic targets including genes involved in extracellular matrix formation. Their work suggests that anti-fibrotic therapy might be a useful therapeutic strategy in addition to gene and cell therapies.

### Dysferlinopathy

Dysferlinopathies are a group of autosomal recessive muscular dystrophies caused by mutations in the *DYSFERLIN* (*DYSF*) gene, involved in fiber repair (Figure 1). Patients exhibit highly elevated serum levels of creatine kinase, severe muscle inflammation and muscle weakness, but phenotypes can vary even between affected family members. *DYSF* delivers some 14 transcripts owing to alternative splicing involving exons 5a, 17 and 40a, as well as the use of two different promoters (Pramono et al., 2009). Expression levels of transcripts encompassing exon40a are quite low, but they deliver a protein with a calpain cleavage site. Upon membrane injury and a subsequent influx of calcium, calpain is activated to cleave this site, releasing a C-terminal mini-dysferlin that plays an important role in membrane repair. Here in this Frontiers Research Topic, Ballouhey et al. showed that calpain-mediated cleavage requires the N-terminal portion of the exon40a-encoded peptide. Moreover, specifically the exon40a-containing transcript 11 plays a key role in the skeletal muscle membrane repair process, protecting the membrane of myoblasts from mechanical stress induced by osmotic shock and facilitating protein vesicle trafficking. These findings suggest that the therapy of dysferlinopathies is possible by including exon 40a in restored transcripts and can tolerate sequence variations existing in the human population. Such detailed understanding of the role of splice variants in the molecular processes of myotubes will provide specific frameworks to apply some of the newer, relatively successful oligonucleotide-based approaches.

## Injury

### Atrophy

Muscle atrophy is characterized by the loss of muscle mass and results in decreased locomotion independence and an increased risk for morbidity and mortality. It arises because of muscle disuse following spinal cord injury, prolonged bed rest or lack of gravity in space flight. Maintenance of skeletal muscle mass relies on two cell populations, the satellite cells or adult muscle stem cells and the fibro-adipogenic progenitor cells (FAPs). FAPs are quiescent mesenchymal precursor cells interstitial to muscle that, like satellite cells, become active after muscle injury (Biferali et al., 2019), whilst satellite cells rebuild the muscle tissue, FAPs have emerged as important facilitators of this process (Figure 1). Here, Parker et al. performed an RNAseq analysis of FAPs from immobilized and non-immobilized limbs. They found that IL-1β, a key component of the senescence-associated secretory phenotype (SASP), was upregulated, as was the cell cycle inhibitor Cdkn2a, a further recognized senescence marker. This suggest that muscle disuse promotes FAP senescence and undermines the ability of FAPs to support muscle maintenance and regeneration, thus leading to atrophy. The authors suggest that their immobilization model also recapitulates some of the molecular and cellular changes occurring in muscle atrophy linked to sarcopenia and cachexia. Together, their work suggests that FAP senescence should be considered as a target for therapy.

Along the same lines, acute muscle injury is accompanied by direct trauma to motor axons. Thus, both muscle and nerve need to regenerate, and new neuromuscular junctions (NMJ) need to be established. Terminal Schwann Cells (TSCs), also known as perisynaptic Schwann cells or teloglia, are non-myelinating Schwann cells at the NMJ (Santosa et al., 2018). They are crucial for the development and maintenance of the NMJ, act as guides to the regenerating neurites and induce new motor boutons and synapses (Son and Thompson, 1995). TSC failure is associated with neuromuscular diseases such as spinal muscular atrophy and amyotrophic lateral sclerosis. Recent studies implicated Sema3a-release from muscle satellite cells in the control of the motor neurite extension towards the synaptic regions of regenerating fibres. However, the interplay between nerve, TSC, satellite cells and muscle fiber, is still unclear. Here, Daneshvar and Anderson activated satellite cells prior to crush injury or cardiotoxin-mediated injury of muscle in which the nerve remains intact. The premature activation of satellite cells uncoupled muscle and NMJ regeneration. They then used qualitative and quantitative approaches to detect the expression of Sema3a, the 2EF-hand calcium-binding protein S100B and the nerve growth factor receptor P75. Their work showed co-expression of these markers in TSC and satellite cells at the site of synchronized NMJ regeneration but suggested reduced co-expression when regeneration of muscle and nerve was uncoupled. Thus, managed dual neuro-muscular regeneration may be an effective approach for restoring muscle after a traumatic injury.

## Aging

### Sarcopenia

Progressive loss of skeletal muscle during aging, called sarcopenia, negatively impacts motility and metabolic homeostasis and is a key factor in the reduced quality of life at old age. It is thought to affect one in three individuals over the age of 60, and more than half of the population over 80 (Haehling et al., 2010). Aging muscle exhibits reduced biosynthesis, slower metabolism, smaller mitochondria, reduced muscle mass and fiber size, and reduce number of organelles and proteins, in addition to reduced regenerative capacity (Yin et al., 2021). Notably, inflammation is highly prevalent in the elderly population. Moreover, aging muscle produces excessive reactive oxygen species (ROS). Here, Chen et al. discussed the physiology of the aging muscle and make the link: ROS leads to oxidative stress in cells, and in turn to oxidative damage including DNA, lipid and protein damage and dysfunctional mitochondria and hence, reduced muscle function Oxidative stress also triggers inflammation, specifically the accumulation of pro-inflammatory cytokines such as IL-6 and TNFα, the stimulation of the phagocytotic action of macrophages and neutrophils and the release of cytotoxic protease, pro-inflammatory cytokines, and ROS, thus exacerbating the oxidative stress as well as promoting catabolic reactions. The authors propose that a combination of exercise, antioxidant and anti-inflammatory therapy are the most effective ways to treat muscle loss during aging.

### Hypoxia

Skeletal muscle plays a pivotal role in body metabolism and the maintenance of protein homeostasis. Aging and systemic disease conditions including cancer, heart failure and obesity, alter muscle integrity by shifting the balance between anabolic and catabolic pathways. The ubiquitously expressed basic-loop-helix transcription factor Atoh8, in mouse also known as Math6, was initially identified as proneural gene (Inoue et al., 2001). It since has been implicated in organogenesis, maintenance of pluripotency and cancer, but was recently linked to the formation of abdominal and intercostal muscles in the embryo (Balakrishnan-Renuka et al., 2014). However, its role in adult muscle was not known. Here, Divvela et al. showed that Atoh8 mouse mutants present with reduced muscle mass, muscle strength and fiber size as well as changes in muscle fiber type akin to the collapse of muscle maintenance found in a condition known as ambient hypoxia. In line with this, expression of Hif1α is upregulated in the knock-out mouse. Using loss and gain of function experiments *in vivo* and *in vitro*, Divvela et al. showed that Atoh8 suppresses the Tgfβ family member Myostatin as well as the cell cycle suppressor Cdkn1a/p21, thus maintaining myoblasts as undifferentiated transit amplifying cells. This effect may be due to an elevation of the AKT/mTOR pathway. The work implicated Atoh8 a novel player in adult muscle homeostasis.

Work in this Research Topic clearly demonstrates that progress towards managing and ameliorating symptoms of muscle disease has been achieved despite the still limited interplay between cell types participating in muscle regeneration. Moreover, novel regulators and mechanisms are still being discovered. However, the exciting prospect is that the understanding of new cellular interactions and new regulators will provide new targets for therapy and will provide innovative approaches to therapy delivery.
